# MRD response in relapsed/refractory FL after obinutuzumab plus bendamustine or bendamustine alone in the GADOLIN trial

**DOI:** 10.1038/s41375-019-0559-9

**Published:** 2019-08-28

**Authors:** Christiane Pott, Laurie H. Sehn, David Belada, John Gribben, Eva Hoster, Brad Kahl, Britta Kehden, Emmanuelle Nicolas-Virelizier, Nathalie Spielewoy, Guenter Fingerle-Rowson, Chris Harbron, Kirsten Mundt, Elisabeth Wassner-Fritsch, Bruce D. Cheson

**Affiliations:** 1 University Hospital Schleswig-Holstein, Kiel, Germany; 2 British Columbia Cancer Agency and the University of British Columbia, Vancouver, BC Canada; 3 Department of Internal Medicine—Haematology, Charles University, Hospital and Faculty of Medicine, Hradec Králové, Czech Republic; 4 Queen Mary University of London, London, UK; 5 Hospital of the Ludwig-Maximilians University, Munich, Germany; 6 Washington University School of Medicine, St Louis, MO USA; 7 University of Lyon, Lyon, France; 8 F. Hoffmann-La Roche Ltd, Basel, Switzerland; 9 Roche, Welwyn Garden City, UK; 10 Georgetown University Hospital, Washington, DC USA

**Keywords:** Haematological cancer, Prognosis

## Abstract

We report assessment of minimal residual disease (MRD) status and its association with outcome in rituximab-refractory follicular lymphoma (FL) in the randomized GADOLIN trial (NCT01059630). Patients received obinutuzumab (G) plus bendamustine (Benda) induction followed by G maintenance, or Benda induction alone. Patients with a clonal marker (t[14;18] translocation and/or immunoglobulin heavy or light chain rearrangement) detected at study screening were assessed for MRD at mid-induction (MI), end of induction (EOI), and every 6–24 months post-EOI/discontinuation by real-time quantitative PCR. At MI, 41/52 (79%) patients receiving G-Benda were MRD-negative vs. 17/36 (47%) patients receiving Benda alone (*p* = 0.0029). At EOI, 54/63 (86%) patients receiving G-Benda were MRD-negative vs. 30/55 (55%) receiving Benda alone (*p* = 0.0002). MRD-negative patients at EOI had improved progression-free survival (HR, 0.33, 95% CI, 0.19–0.56, *p* < 0.0001) and overall survival (HR, 0.39, 95% CI, 0.19–0.78, *p* = 0.008) vs. MRD-positive patients, and maintained their MRD-negative status for longer if they received G maintenance than if they did not. These results suggest that the addition of G to Benda-based treatment during induction can significantly contribute to the speed and depth of response, and G maintenance in MRD-negative patients potentially delays lymphoma regrowth.

## Introduction

Relapsed disease remains a significant challenge in the treatment of indolent non-Hodgkin lymphoma (NHL) [1]. Early identification of patients who are at high risk of relapse may be important for treatment optimization. In addition to imaging techniques, assessment of minimal residual disease (MRD) has emerged as potentially important for the detection of residual tumor cells, and thus the evaluation of treatment efficacy and long-term prognosis in this patient population [2–9]. Achieving MRD response has been associated with improved outcome in patients with follicular lymphoma (FL), independent of clinical remission status, and pretreatment patient characteristics [8]. However, previous studies on the use of MRD assessment in NHL have predominantly been in first-line patient populations, and data on MRD in patients with relapsed/refractory disease are limited.

Obinutuzumab (G) is a glycoengineered, type II anti-CD20 antibody with enhanced biological activity when compared with type I antibodies, such as rituximab [10, 11]. G is approved for treatment of chronic lymphocytic leukemia (CLL; first-line in combination with chlorambucil) and FL (first-line in combination with chemotherapy, followed by G maintenance; relapsed/rituximab-refractory disease in combination with bendamustine [Benda], followed by G maintenance) [12, 13].

GADOLIN (NCT01059630) was an international, open-label, multicenter, and randomized phase 3 trial evaluating the efficacy and safety of G plus Benda induction followed by G maintenance (G-Benda arm) vs. Benda induction alone (Benda arm) in patients with rituximab-refractory indolent NHL (iNHL; predominantly FL) [14]. The study showed a statistically significant improvement in progression-free survival (PFS) and overall survival (OS) in the G-Benda arm compared with the Benda arm [14–16]. No difference was observed between treatment arms in overall or complete response (CR) rates at the end of induction (EOI) [14–16].

Using updated efficacy data with a median follow up of 31.8 months, this paper reports the results of a preplanned MRD assessment in patients with FL enrolled in GADOLIN. The main objectives of the analysis were: (1) to evaluate the depth and time course of the MRD response to G-Benda or Benda by assessing MRD during induction (mid-induction; MI) and at EOI, and throughout G maintenance (G-Benda arm) or follow up (Benda arm); and (2) to assess the association between EOI MRD status and PFS and OS.

## Methods

### Data sharing statement

Qualified researchers may request access to individual patient level data through the clinical study data request platform (www.clinicalstudydatarequest.com). Further details on Roche’s criteria for eligible studies are available here (https://clinicalstudydatarequest.com/Study-Sponsors/Study-Sponsors-Roche.aspx). For further details on Roche’s Global Policy on the Sharing of Clinical Information and how to request access to related clinical study documents, see here (https://www.roche.com/research_and_development/who_we_are_how_we_work/clinical_trials/our_commitment_to_data_sharing.htm).

### Study design and patients

The GADOLIN study design and patient population has been described in full elsewhere [14]. In brief, patients aged 18 years or older with rituximab-refractory, histologically documented CD20 + iNHL were randomly assigned (1:1) to six cycles of G (cycle 1: 1000 mg on days 1, 8, and 15; cycles 2–8: 1000 mg on day 1) plus Benda (cycles 1–6: 90 mg/m^2^ on days 1 and 2; G-Benda arm) or Benda alone (cycles 1–6: 120 mg/m^2^ on days 1 and 2; Benda arm). Randomization was stratified according to iNHL subtype (FL or other), refractory type (rituximab monotherapy or rituximab plus chemotherapy), number of previous therapies (two or more, or less than two), and geographic region. Patients in the G-Benda arm with no evidence of progression at EOI (i.e., those with CR, partial response [PR], or stable disease [SD]) received G maintenance (1000 mg) every 2 months for 2 years. The study was conducted in accordance with the Declaration of Helsinki and the International Conference on Harmonisation guidelines for Good Clinical Practice. The protocol was approved by the ethics committees of the participating centers. Written consent for MRD assessment was provided by patients.

### Definition of MRD status and analysis time points

MRD status was evaluated at screening in peripheral blood (PB) and bone marrow (BM), at MI (cycle 4, day 1) in PB, at EOI in PB and BM, and at 6-monthly intervals during maintenance (G-Benda arm) or follow up (Benda arm) in PB up to 24 months post-EOI or treatment discontinuation. MRD status of a sample was defined as positive (MRD-positive) if one of the real-time quantitative polymerase chain reaction (RQ-PCR) and subsequent nested PCR assays produced a specific PCR signal according to the applied criteria and quality checks. MRD status of a sample was defined as negative (MRD-negative; also sometimes referred to as MRD undetectable) if RQ-PCR and subsequent nested PCR produced no specific PCR signal in a sample with at least 10^4^ control gene copies (albumin control gene). MRD status at a time point was defined as positive if at least one of all evaluable samples (PB or BM) was positive by RQ-PCR confirmed by nested PCR. In patients for whom both PB and BM samples were available, the higher MRD value was used for calculation of quantitative MRD values and assessment. Concordance of PB and BM results was assessed, i.e., results where both samples were negative (undetectable) and where both were positive (including positive but unquantifiable).

Assessments during maintenance/follow up were conducted at 6, 12, and 18 months after EOI, and at the final (∼28 days after the 24-month visit) or early termination/discontinuation visit. Only patients without disease progression at EOI were assessed for MRD during maintenance/follow up. Samples collected at or subsequent to documented clinical relapse were not included in the statistical analysis.

### MRD assessment

MRD assessment was based on detection of clonal markers in PB and BM aspirate samples at screening in patients with FL; patients with other types of iNHL were not included in this analysis. Samples were analyzed centrally in a reference laboratory in Kiel, Germany.

The diagnostic PB and BM samples were screened initially by consensus PCR (modified according to van Dongen et al.) [17] to detect a t(14;18) translocation and/or clonal immunoglobulin heavy chain (IGH) or light chain (IG kappa [IGK]) rearrangement suitable for MRD assessment. In patients with a detectable clonal marker, sequencing of clonal IG rearrangements was done for the design of allele-specific oligonucleotides (ASO) for RQ-PCR. All assays were designed with a sensitivity of ≤10^−5^, but were accepted for analysis when a sensitivity of ≤10^−4^ was reached. In case of two available markers, the most sensitive marker was chosen for MRD analysis. MRD quantification was performed as described previously [18, 19]. For determining the quantitative MRD levels, target copy numbers were related to the number of target copies of tenfold serial dilutions of cell lines or plasmids used for quantification of the respective rearrangement. In patients for whom no marker could be detected in PB or BM, identification of the clonal rearrangement from formalin-fixed, paraffin-embedded tissue (FFPET; lymph nodes [LNs]) was attempted.

Standards for quantification by RQ-PCR were obtained by different approaches. For patients with a t(14;18) translocation (major breakpoint region [MBR], 3′MBR, minor cluster region [MCR] and 5′MCR), either cell lines (DOHH2, SC1, K231) or two cloned plasmids bearing all known t(14;18) breakpoint sites of MBR, 3′MBR, MCR, 5′MCR cloned into a pEN08H vector were used to establish standard curves for quantification. A forward primer and probes were placed in chromosome 18 in combination with a reverse primer placed in the consensus JH region on chromosome 14. This was designed in such a way that RQ-PCR products did not exceed a maximum of 200 base pairs in size, to prevent differences in amplification efficiency. The plasmids had been standardized for sensitivity and reproducibility in multiple quality control rounds of the EuroMRD consortium.

For patients with a clonal IG rearrangement, the specific IGH or IGK rearrangement was cloned, and ASO primers were designed for ASO RQ-PCR.

Cloning was performed using the TOPO TA cloning Kit (Thermo Fisher Scientific, MA, USA). Quantification by plasmid standards was conducted using a modified protocol according to Gimenez et al. [20]. MRD levels were given as a fraction of number of FL cells per total number of mononuclear BM or PB cells analyzed per PCR assay. All RQ-PCR data were evaluated according to EuroMRD consortium guidelines [19] to establish sensitivity and quantitative range for all patients and to measure tumor load in each sample. For confirmatory reasons, all samples analyzed by RQ-PCR were additionally analyzed by nested PCR adapted from the published literature [21, 22].

### Statistical analysis

MRD status at MI, EOI, and during maintenance or follow up was tabulated against treatment arm, and the association was tested using Fisher’s Exact test. The association of PFS and OS with MRD status at EOI was determined using a landmark analysis measuring survival time from the date of the EOI MRD sample (to avoid ascertainment bias) in patients without progression at EOI. Differences between patient subgroups were visualized using Kaplan–Meier plots and analyzed with Cox proportional hazards models. The data cut off for the current analysis was April 1, 2016. Statistical analyses were conducted using SAS v9.2.

## Results

### MRD marker identification

From April 15, 2010 to January 24, 2015, 335 patients with FL were enrolled in the GADOLIN study. From the 319 patients with PB and/or BM samples available at baseline (baseline-evaluable population), 554 diagnostic samples were screened (313 PB, 189 BM, 52 LNs). Clonal markers were detected in 228/319 (71%) patients. In 22 of these patients, clones were detected in LN FFPET. Two of these were subsequently detected in BM and seven in PB; the remaining 13 could not be detected in either PB or BM and so no MRD analysis was performed in follow-up samples.

A detectable MBR, 3′MBR or MCR t(14,18) translocation was identified in 144/228 (63%) patients, with this being the only marker identified in 63 patients. A clonal IG rearrangement was detected in 165/228 (72%), with this being the only marker identified in 84 patients. Both markers were present in 81/228 (36%) patients (Table 1).

**Table 1:** Frequency of *BCL2* and Ig-based MRD markers for all patients

Patients with molecular marker	*t*(14;18) only	IgH only	Both marker positive
228	63	84	81

In total, 166/319 patients (52%) had an RQ-PCR assay fulfilling the sensitivity criteria (≤10^−4^), 90% of those achieving a sensitivity of 10^−5^. In the majority of cases, a t(14;18) translocation-based RQ-PCR assay was used (62% MBR/3′MBR breakpoint, 8% MCR/5′MCR breakpoint) and in 30%, IGH/IGK rearrangements served as an MRD target.

**Fig. 1:**
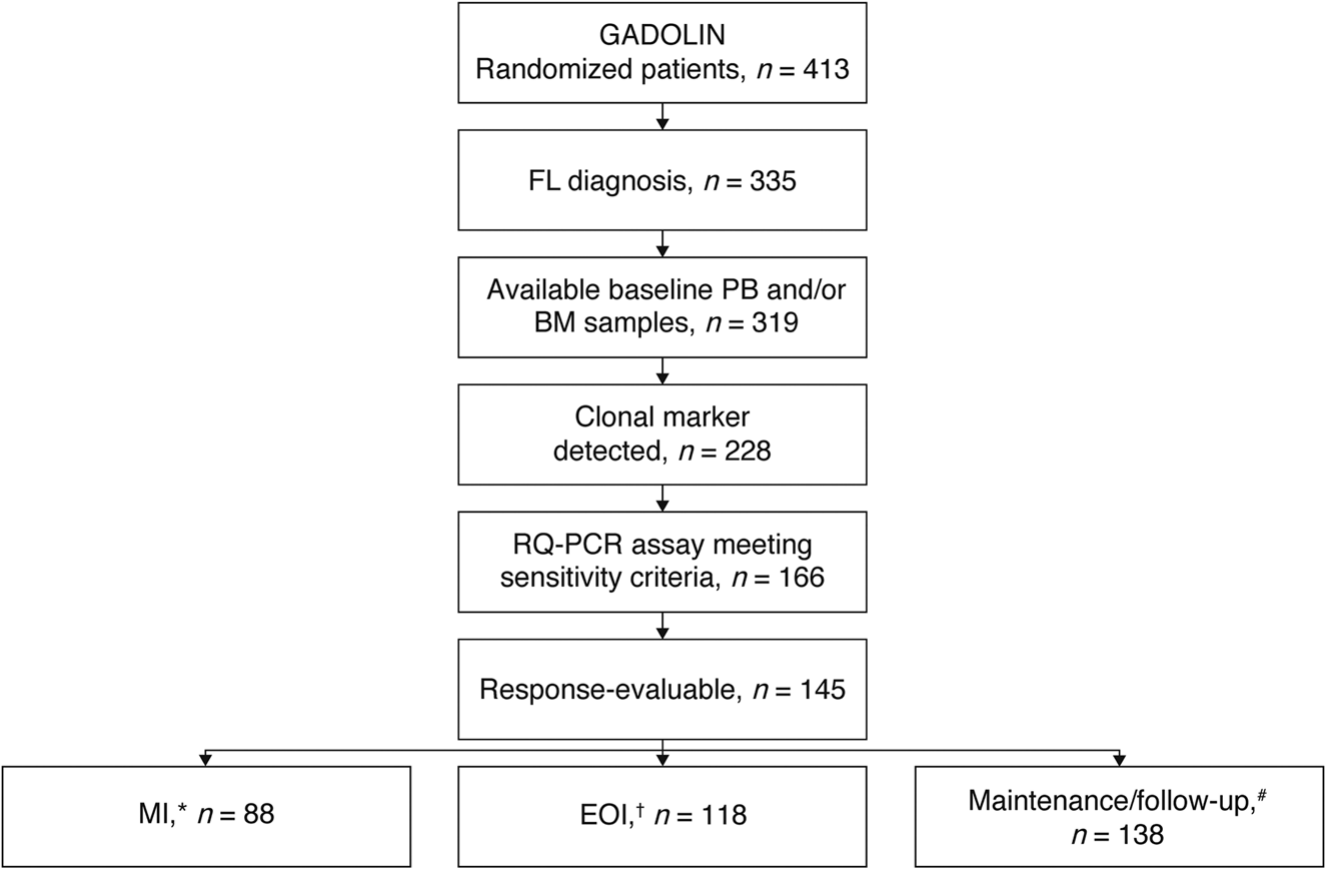
MRD analysis population. The asterisk indicates the PB sample available at MI for assessment of MRD response kinetics; the dagger indicates the PB and/or BM sample available at EOI for MRD response assessment (MRD-evaluable population); the hash symbol indicates the patients with ≥1 MRD sample at EOI (in PB/BM) and/or during maintenance and follow up (PB). BM bone marrow, EOI end of induction, FL follicular lymphoma, MI mid-induction, MRD minimal residual disease, PB peripheral blood, and RQ-PCR real-time quantitative polymerase chain reaction

Overall, 145 patients were evaluable for MRD response assessment at any of the timepoints: MI (*n* = 88), EOI (*n* = 118), or had at least one MRD assessment during maintenance and follow up (*n* = 138). (MRD-response evaluable population, Fig. 1).

A clonal marker was detected more frequently in patients with a higher incidence of Ann Arbor stage IV disease at diagnosis, higher Follicular Lymphoma International Prognostic Index (FLIPI) score at initial diagnosis, and more frequent BM and extranodal involvement at baseline (Table 2), with a slightly higher proportion of patients in the G-Benda treatment arm displaying these worse prognostic factors. Overall, this resulted in poorer PFS in patients with a detectable marker (Supplementary Fig. S1) compared with those without a detectable clonal marker, reflecting a higher tumor load in this patient population. Time from last treatment to randomization was similar in the two groups (median 3.7 vs. 3.9 months in patients with and without a detectable clonal marker, respectively). Other demographic parameters, including age and sex, were well balanced between the populations, and when compared with the overall GADOLIN population [14].

**Table 2:** Demographics and baseline disease characteristics for patients with or without a detectable clonal marker, and all baseline-evaluable patients

	No marker detected	Marker detected, with/without RQ-PCR assay	Total
	(*n* = 91)	(*n* = 228)	(*n* = 319)
Median age, years (range)	63 (34–87)	63 (34–87)	63 (34–87)
Male, *n* (%)	49 (53.8)	129 (57)	178 (55.8)
ECOG PS, *n* (%)	(*n* = 91)	(*n* = 227)	(*n* = 318)
0–1	89 (97.8)	214 (94)	303 (95.3)
2	2 (2.2)	13 (6)	15 (4.7)
Ann Arbor stage,^a^ *n* (%)			
**I**	**11 (12.9)**	**7 (3)**	**18 (6.0)**
II	13 (15.3)	22 (10)	35 (11.6)
**III**	**30 (35.3)**	**45 (21)**	**75 (24.8)**
**IV**	**31 (36.5)**	**143 (66)**	**174 (57.6)**
Unknown	6	11	17
FLIPI, 1 adverse factors risk category,^a^ *n* (%)	(*n* = 91)	(*n* = 227)	(*n* = 318)
**Low (0–1)**	**35 (40.7)**	**41 (19)**	**76 (24.9)**
Intermediate (2)	26 (30.2)	78 (36)	104 (34.1)
**High (≥3)**	**25 (29.1)**	**100 (46)**	**125 (41.0)**
Unknown	5	8	13
Bone marrow involvement,^a^ *n* (%)	(*n* = 87)	(*n* = 218)	(*n* = 305)
**Positive**	**9 (10.3)**	**83 (38)**	**92 (30.2)**
**Negative**	**74 (85.1)**	**127 (58)**	**201 (65.9)**
Insufficient sample	3 (3.4)	5 (2)	8 (2.6)
Other	1 (1.1)	3 (1)	4 (1.3)
Extranodal involvement,^a^ *n* (%)			
Yes	37 (46.3)	120 (55)	157 (52.9)
No	43 (53.8)	97 (45)	140 (47.1)
Unknown	11	11	22
Time from initial diagnosis to randomization (months)^a^			
Mean (SD)	50.6 (41.3)	51.9 (53.9)	51.5 (50.6)
**Median (range)**	**39.8 (3.8–215.6)**	**34.6 (3.1–384.8)**	**36.2 (3.1–384.8)**
Time from last regimen to randomization (months)^a^			
**Mean (SD)**	**5.8 (6.03)**	**8.0 (13.3)**	**7.4 (11.7)**
Median (range)	3.7 (0.7–37.5)	3.9 (0.7–128.4)	3.8 (0.7–128.4)

*ECOG* Eastern Cooperative Oncology Group, *FLIPI* Follicular Lymphoma International Prognostic Index, *PS* performance status, *RQ-PCR* real-time quantitative polymerase chain reaction, *SD* standard deviation

^a^Differences ≥10% between groups are in bold

### MRD status and kinetics

At study entry, 202/313 (65%) PB and 105/189 (55%) BM samples had a detectable clonal marker. Quantitative assessment of circulating lymphoma cells (CLC) revealed a median level of 0.5% lymphoma cells in PB at screening. In BM, a median infiltration of 2% FL cells was determined. Low-level CLC or BM infiltration below the limit of quantification (<10^−4^) was present in 33/164 (20%) PB samples and 21/94 (22%) BM samples.

For 91 patients who had RQ-PCR results for both PB and BM samples at screening, RQ-PCR values for the two samples correlated, and there was a high level of concordance between the PB and BM results for these patients (*r*^2^ = 0.71) (Supplementary Fig. S2). Overall, 78 of 91 paired diagnostic samples were concordantly positive or positive below the limit of quantification and six were concordantly negative. Discordant results occurred in only seven paired samples; in five of these, the positive samples demonstrated low-level positivity below the quantitative range. Of the two discordant cases, one was positive in BM and negative in PB, the other one positive in PB and negative in BM.

**Fig. 2:**
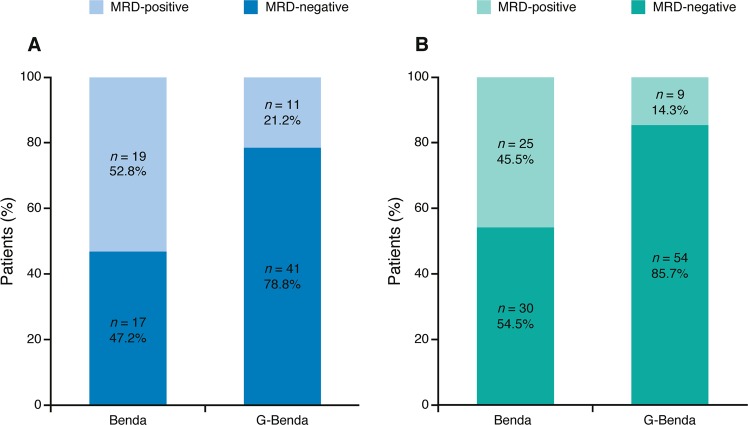
MRD status at MI in PB (**a**) and at EOI in PB and/or BM (**b**). Benda bendamustine, BM bone marrow, EOI end of induction, G obinutuzumab, MI mid-induction, MRD minimal residual disease, and PB peripheral blood

**Fig. 3:**
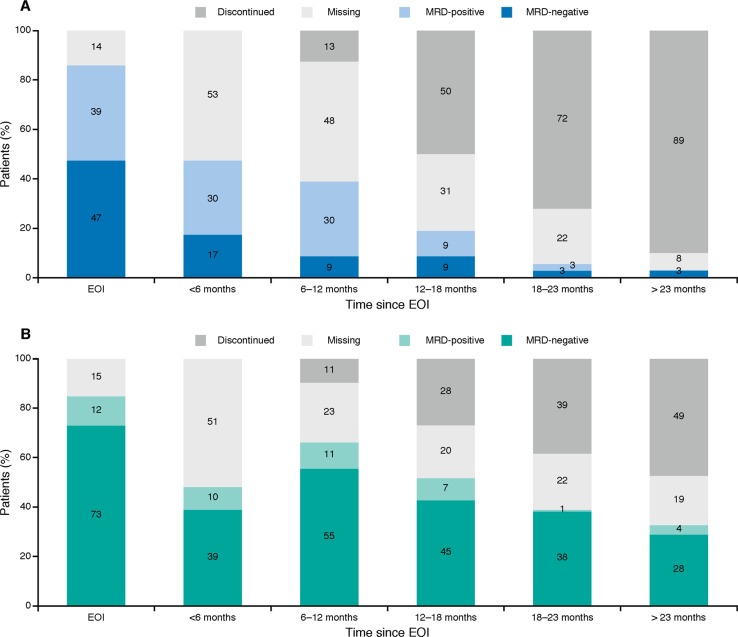
MRD status in blood at the end of induction, and throughout G maintenance or follow up in the Benda arm (**a**) and in the G-Benda arm (**b**). Benda bendamustine, BM bone marrow, EOI end of induction, G obinutuzumab, MRD minimal residual disease, and PB peripheral blood

MRD negativity in PB occurred early during induction and was more rapid and frequent in the G-Benda treatment arm; 41/52 (78.8%) patients in the G-Benda arm were MRD-negative at MI vs. 17/36 (47.2%) patients in the Benda arm (*p* = 0.0029; Fig. 2a). At EOI, more patients had MRD responses in PB and/or BM, with 54 of 63 (85.7%) patients in the G-Benda arm vs. 30 of 55 (54.5%) patients in the Benda arm (*p* = 0.0002; Fig. 2b). During maintenance in the G-Benda arm and follow up in the Benda arm, missing samples and increasing discontinuation rates impacted the number of MRD evaluable patients. Nevertheless, the majority of MRD-evaluable patients (55/66; 83%) in the G-Benda arm were MRD-negative compared with only 9 of 39 (23%) patients in the Benda arm (who received no maintenance treatment) in the 6–12-month sampling window (Fig. 3). Of note, over the 2-year post induction period, the rate of discontinuation was much higher in the Benda arm compared with the G-Benda arm (89 vs. 49% after more than 23 months), and a much lower proportion of patients remained MRD-negative (3% in the Benda arm vs. 28% in the in the G-Benda arm).

### Association between MRD status and outcome

**Fig. 4:**
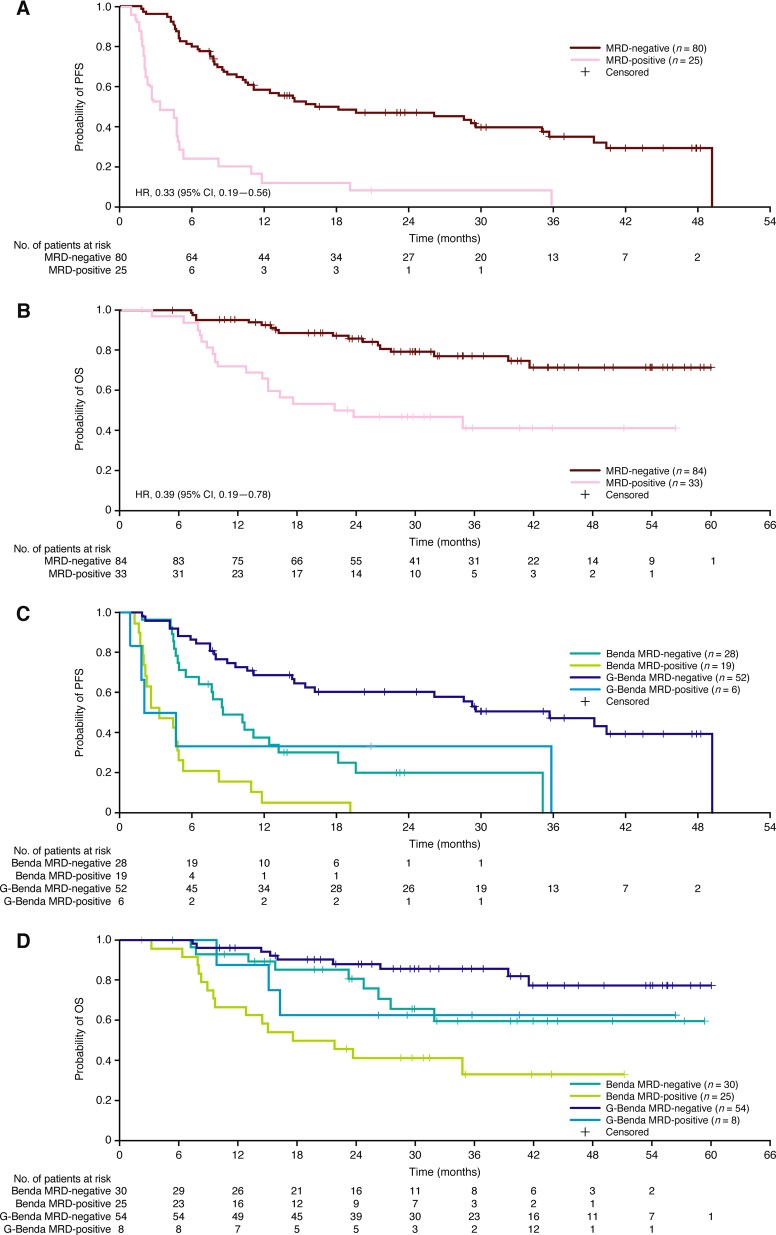
PFS (**a**) and OS (**b**) by MRD status at EOI in PB and/or BM, and PFS (**c**) and OS (**d**) by MRD status at EOI in PB and/or BM and by treatment arm in patients without progression at EOI. Benda bendamustine, BM bone marrow, EOI end of induction, G obinutuzumab, MRD minimal residual disease, PB peripheral blood, and PFS progression-free survival

Of the patients with a CR or PR from both treatment arms, those who were MRD-negative in PB and/or BM at EOI had longer subsequent PFS (HR, 0.33; 95% CI, 0.19–0.56) and OS (HR, 0.39; 95% CI, 0.19–0.78) compared with those who were MRD-positive (Fig. 4a, b). MRD-positive patients in the G-Benda and Benda arms had similar unfavorable median PFS values, i.e., 3.3 months (95% CI, 2.20–8.18) and 3.3 months (1.74–not reached), respectively (Fig. 4c). For MRD-negative patients, those in the Benda arm had a shorter PFS (median, 8.54 months; 95% CI, 6.54–18.10) than those in the G-Benda arm (median, 35.71 months; 95% CI, 16.16–not reached). The HRs for PFS in each arm (MRD-negative relative to MRD-positive) were 0.29 (95% CI, 0.11–0.76) for G-Benda and 0.32 (95% CI, 0.17–0.60) for Benda. For OS, the HRs were 0.37 (95% CI, 0.01–1.38) and 0.39 (95% CI, 0.17–0.89), respectively. MRD-positive patients in the Benda arm tended to relapse quickly, as did MRD-positive patients in the G-Benda group, four of whom progressed within the first 6 months after EOI. However, it should be noted that patients in the G-Benda arm received G maintenance after EOI, while those in the Benda arm did not.

Achievement of MRD negativity at EOI was associated with clinical remission (CR or PR) (Table 3); however, there were five patients who had a CR but were MRD-positive at EOI. In three of these patients, the CR was short-lasting, with disease progression recorded 2–5 months later. In the other two patients, whose CR responses lasted for at least two further years, only low-level MRD below the limit of quantification had been detectable in BM at EOI.

**Table 3:** Correlation of clinical response with MRD status at EOI

	CR (*n* = 23)	PR (*n* = 76)	SD (*n* = 3)	PD (*n* = 9)	Missing	Total
MRD-positive, *n* (%)	5 (21.7)	16 (21.1)	2 (66.7)	7 (77.8)	4	34
MRD-negative, *n* (%)	18 (78.3)	60 (78.9)	1 (33.3)	2 (22.2)	3	84

*CR* complete response, *EOI* end of induction, *MRD* minimal residual disease, *PD* progressive disease, *PR* partial response, *SD* stable disease

The independent value of MRD response for both PFS and OS after the EOI was explored in a multivariate model, which included treatment and baseline assessments for bulky disease, extranodal involvement, FLIPI status, the number of previous lines of therapy, double refractory status, and sex (Supplementary Table S1). The model results are summarized using a likelihood ratio test, assessing the additional influence of each variable on the overall model, given all the other variables. For PFS, MRD status at EOI was found to be strongly predictive, along with treatment arm and FLIPI status. For the OS model, the same variables had the greatest predictive value, although to a lesser extent than for PFS. The HR for MRD status at EOI, adjusted for treatment and baseline factors, was 0.214 for PFS and 0.317 for OS.

## Discussion

We prospectively analyzed MRD in the context of the GADOLIN trial to determine response to G-Benda and Benda treatment arms at the molecular level, and to investigate whether MRD status after induction therapy at relapse has an impact on prognosis.

There was an imbalance in disease characteristics at baseline between patients with and without detectable clonal markers, with greater prevalence of poor prognostic factors in the patients with detectable clonal markers, which was reflected by a shorter PFS. Consequently, the population analyzed for MRD response was not fully representative of the complete GADOLIN study sample.

In the current study, a significantly greater proportion of patients receiving G-Benda achieved MRD negativity at EOI compared with patients receiving Benda alone. Remarkably, the majority of evaluable patients in the G-Benda arm had already achieved MRD negativity by MI, suggesting rapid response kinetics, and demonstrating that the combination of G with Benda significantly contributes to the depth and speed of response during induction treatment. MRD response at EOI was also associated with achievement of clinical PR/CR at EOI (Table 3). These findings suggest that MRD assessment is not only a sensitive tool for response assessment, but also allows early identification of clinical responders.

Given that patients in the GADOLIN trials were previously treated with (chemo)immunotherapy (median two prior treatments), and were in part rituximab-refractory, it is remarkable that the rate of MRD negativity achieved at EOI in the G-Benda arm (85.7% of the MRD-evaluable population) is comparable with that reported in a study of patients with FL treated first-line with rituximab plus chemotherapy (85%) [23]. However, in first-line treatment of patients with FL and high tumor burden, immunochemotherapy with G (GALLIUM trial) achieved even higher rates of MRD negativity at EOI, i.e., 92% in patients receiving G-based therapy and 85% in those on rituximab-based therapy [24].

Patients who were MRD-positive at EOI had a poor prognosis, irrespective of treatment arm, while an MRD-negative status at EOI was significantly associated with improved PFS and OS, regardless of treatment. However, patients in the G-Benda arm achieving clinical and MRD response and receiving G-maintenance appear to have the most favorable outcome. In contrast, patients achieving clinical CR but no MRD response after G-Benda relapsed quickly within the first 6 months after EOI despite G-maintenance, demonstrating that MRD response is critical and predictive for disease control by maintenance treatment.

Maintenance treatment with G appeared to sustain the MRD response in patients who achieved MRD-negative status in the G-Benda arm. In contrast, MRD-negative patients in the Benda arm who did not receive maintenance were found to relapse earlier and convert back to MRD positivity. This seems somehow contradictory to data in first-line treatment of FL patients, where long-term prognosis is excellent when patients achieve MRD response after induction, independent of treatment [25].

Beside the fact that it is difficult to compare data on first-line and relapse treatment, it can be assumed that the residual lymphoma load below the limit of detection of RQ-PCR is probably higher in the less effective Benda arm compared with the antibody combination treatment, resulting in a faster lymphoma regrowth kinetics. However, more importantly, MRD-negative G-Benda patients received ongoing active maintenance treatment, which contrasts with the MRD-negative Benda cohort. Considering the concept of MRD response as a surrogate marker for sensitivity to treatment, it is expected that treatment intervention with an efficient maintenance therapy would affect outcome.

Although these results should be interpreted with some caution due to the low patient numbers in the Benda arm during follow up, our observation supports the use of G maintenance in sustaining MRD negativity and repressing regrowth of the lymphoma clone. Maintaining MRD negativity throughout rituximab maintenance has previously been associated with improved PFS [22].

In the primary GADOLIN analysis, no substantial differences were observed in computed tomography (CT)-based CR rates (11% G-Benda vs. 12% Benda) or overall response rates (69% G-Benda, 63% Benda) at staging after induction, although PFS and OS were improved in the G-Benda arm relative to the Benda arm [14]. However, this was clearly the case when applying MRD for response assessment; a significant difference in MRD response rates was detectable between the two treatment arms. The association between MRD negativity and improved PFS and OS in the current study may suggest that MRD assessment is a more sensitive response measure than standard CT-based response assessment [15, 24], although this is limited to the patient population with a detectable circulating clonal marker at baseline.

In patients with relapsed/refractory FL, a CT-based response assessment of PR may be based on the finding of residual LN enlargement. However, this enlargement may not reflect residual disease, but rather residual fibrotic tissue [26] or slowly responding disease, providing a possible explanation for why a number of patients in the current study were assessed as having a PR, despite being MRD-negative at EOI (*N* = 60).

A recent study of MRD assessment in patients with CLL, demonstrated that the MRD status assessed in PB in responding patients according to the 2008 International Workshop on Chronic Lymphocytic Leukemia criteria has greater predictive value than the prognostic impact of clinical complete or partial remission and allows for improved PFS prediction in patients who achieve a PR or CR [27].

Furthermore, a similar analysis in patients with FL showed that PFS did not differ significantly between MRD-negative patients with either a CR or a PR [8]. Consequently, these results suggest that MRD-negativity may have better prognostic value for long-term outcome than clinical response.

The current study demonstrated a lower frequency of marker detection in PB and BM at study entry compared with first-line FL treatment in the GALLIUM trial [24]. This may be due to recent prior rituximab and/or chemotherapy treatment (median 4 months) in the GADOLIN population, and consequent B-cell depletion. Median time from last treatment to randomization was slightly longer in the clonal marker-detectable vs. the nondetectable population, although it should be noted that this difference was not significant. As the median level of CLC is only 0.5% in PB, a greater number of screening BM samples with a higher level of FL cell infiltration might have improved the rate of marker detection. For future clinical trials with prospective MRD assessment, systematic analysis of DNA extracted from FFPET (LN) could be of value to identify an MRD marker in a higher proportion of patients. However, of the 22 patients in our study whose marker was not identified from peripheral samples but was identified in LN tissue, subsequent analysis of PB and/or BM samples at screening by allele specific RQ-PCR detected lymphoma cells in only nine patients. This suggests that in some of these patients, relapse is mainly located in the involved LN rather than in other compartments. Here, like in diffuse large B-cell lymphoma, the analysis of circulating tumor DNA as a fragment of cell-free DNA might be much more informative for MRD assessment than tracing CLC by genomic DNA analysis [28].

Positron emission tomography (PET) scanning was not used in the GADOLIN trial, as information on its utility in iNHL was scarce at the time of study design;[14] however, PET is now recommended for NHL disease staging and assessment of response to treatment due to its superior accuracy over standard CT [15]. Therefore, it may be useful to combine both PET and MRD in response assessment, as the techniques address different compartments for residual lymphoma cells. A combined PET and MRD response after induction might be also used as endpoint in clinical trials to reduce treatment in patients with excellent prognosis. A recent analysis of 41 patients with FL who were treated with rituximab plus chemotherapy as part of the FOLL05 trial did not find a strong correlation between PET and MRD, but suggested that they could be used as complementary techniques at the end of therapy [29].

In the same way that metabolic response is used to stratify patients for radiation treatment in Hodgkin’s lymphoma and aggressive lymphomas, MRD response could be used to stratify patients for different maintenance strategies, including experimental treatment with novel drugs in MRD-positive patients.

In conclusion, the current analysis of MRD in patients with rituximab-refractory FL enrolled in the GADOLIN trial found improved MRD negativity rates in patients receiving G-Benda vs. Benda alone. MRD negativity at EOI was associated with improved PFS and OS, with this being more pronounced in patients who received maintenance therapy. The findings support the notion that MRD status at EOI is a sensitive marker of the efficacy of G-based treatment in the setting of relapsed/refractory FL. In addition, the findings of the current study, and of others such as the GALLIUM study, support the use of MRD assessment as part of routine clinical practice; however, further research is needed to determine the appropriate management strategy in patients with MRD positivity.

## Supplementary information

Supplementary Information
