# Supplementary material for: MRD response in relapsed/refractory FL after obinutuzumab plus bendamustine or bendamustine alone in the GADOLIN trial
Source: Leukemia. 2019 Aug 28;34(2):522–32. doi: 10.1038/s41375-019-0559-9 (PMC7214251; doi:10.1038/s41375-019-0559-9)
Supplement: Supplementary file 1 — Supplementary Information [file 41375_2019_559_MOESM1_ESM.docx]

**Supplementary Information
Supplementary Figure S1. PFS by detection of a clonal marker at baseline.**


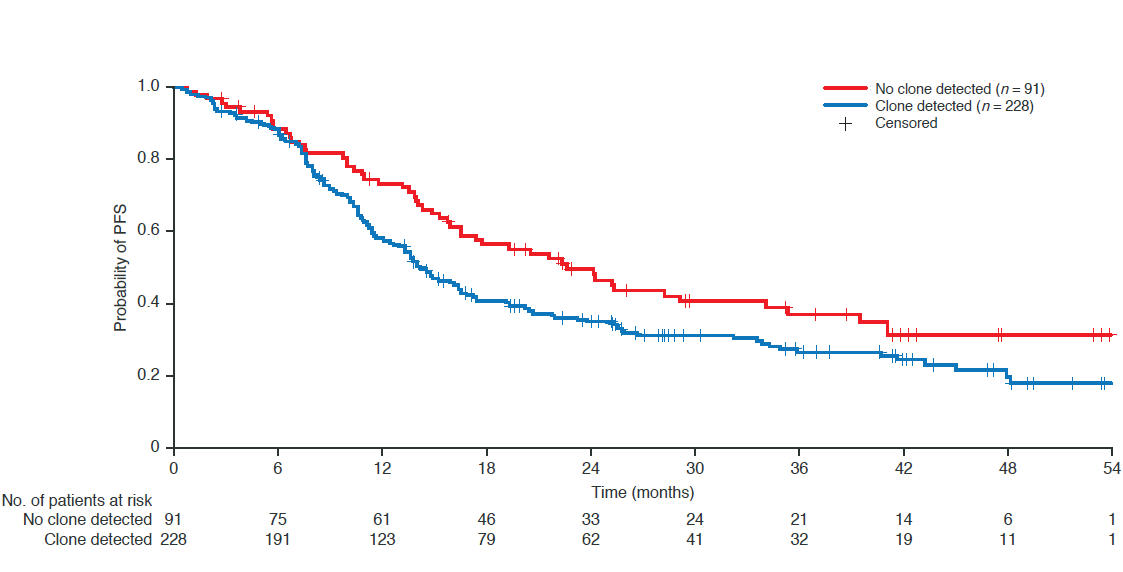


PFS, progression-free survival

**Supplementary Figure S2. Correlation of circulating lymphoma cells in PB with BM infiltration in 91 corresponding diagnostic PB and BM samples.**


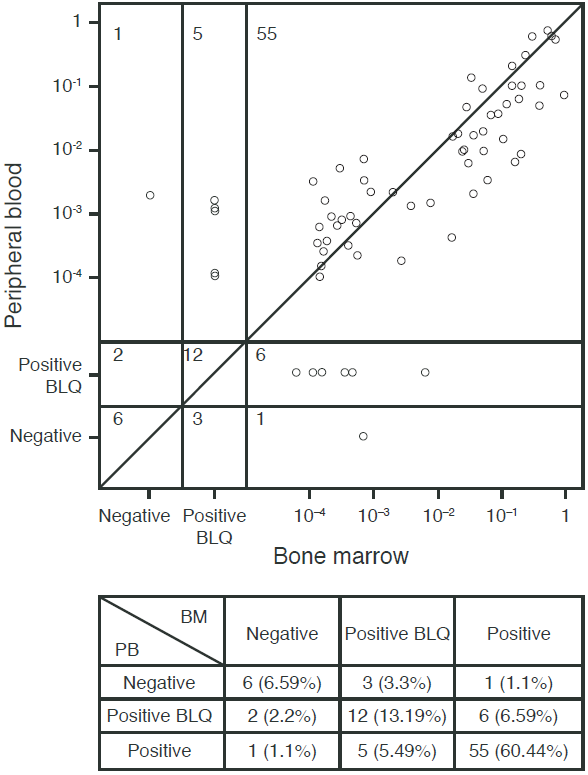


BLQ, below the limit of quantification; BM, bone marrow; MRD, minimal residual disease; PB, peripheral blood

**Supplementary Table S1. Multivariate analysis to determine the independent predictive value of MRD for PFS and OS**

| Variable | Levels | Degrees of freedom  for LRT | PFS | | OS | |
| --- | --- | --- | --- | --- | --- | --- |
|  |  |  | LRT | p-value | LRT | p-value |
| Treatment | G-B, B | 1 | 11.68 | <0.001 | 2.50 | 0.11 |
| Bulky disease at baseline | Yes, no | 1 | 1.83 | 0.18 | 0.00 | 0.96 |
| Extranodal involvement at baseline | Yes, no, unknown | 2 | 5.44 | 0.066 | 1.58 | 0.45 |
| FLIPI at baseline | High, intermediate, low, unknown | 3 | 11.78 | 0.008 | 11.21 | 0.011 |
| Number of previous lines | 1, 2, ≥3 | 2 | 1.05 | 0.59 | 4.77 | 0.092 |
| Double refractory status | No, yes | 1 | 0.16 | 0.69 | 0.17 | 0.68 |
| Sex | Male, female | 1 | 4.35 | 0.037 | 0.35 | 0.56 |
| MRD Status at EOI | Negative, positive | 1 | 18.97 | <0.0001 | 6.52 | 0.011 |

B, bendamustine; EOI, end of induction; FLIPI, Follicular Lymphoma International Prognostic Index; G, obinutuzumab; LRT, likelihood ratio test statistic; MRD, minimal residual disease; OS, overall survival; PFS, progression-free survival
